# Hidden biofilms in a far northern lake and implications for the changing Arctic

**DOI:** 10.1038/s41522-017-0024-3

**Published:** 2017-07-06

**Authors:** Vani Mohit, Alexander Culley, Connie Lovejoy, Frédéric Bouchard, Warwick F. Vincent

**Affiliations:** 1 Département de biologie & Centre d’études nordiques (CEN), Université Laval, Québec, QC G1V 0A6 Canada; 2 Département de biochimie, de microbiologie et de bio-informatique, & Centre d’études nordiques (CEN), Université Laval, Québec, QC G1V 0A6 Canada; 3 Institut de biologie intégrative et des systèmes (IBIS), Université Laval, Québec, QC G1V 0A6 Canada; 4 Takuvik, Unité Mixte Internationale (UMI 3376) Université Laval (Canada) & Centre National de la Recherche Scientifique (France), Québec, QC GIV 0A6 Canada; 5 Centre Eau Terre Environnement, Institut national de la recherche scientifique (INRS), Centre d’études nordiques (CEN), Québec, QC G1K 9A9 Canada

## Abstract

Shallow lakes are common across the Arctic landscape and their ecosystem productivity is often dominated by benthic, cyanobacterial biofilms. Many of these water bodies freeze to the bottom and are biologically inactive during winter, but full freeze-up is becoming less common with Arctic warming. Here we analyzed the microbiome structure of newly discovered biofilms at the deepest site of a perennially ice-covered High Arctic lake as a model of polar microbial communities that remain unfrozen throughout the year. Biofilms were also sampled from the lake’s shallow moat region that melts out and refreezes to the bottom annually. Using high throughput small subunit ribosomal RNA sequencing, we found more taxonomic richness in Bacteria, Archaea and microbial eukaryotes in the perennially unfrozen biofilms compared to moat communities. The deep communities contained both aerobic and anaerobic taxa including denitrifiers, sulfate reducers, and methanogenic Archaea. The water overlying the deep biofilms was well oxygenated in mid-summer but almost devoid of oxygen in spring, indicating anoxia during winter. Seasonally alternating oxic-anoxic regimes may become increasingly widespread in polar biofilms as fewer lakes and ponds freeze to the bottom, favoring prolonged anaerobic metabolism and greenhouse gas production during winter darkness.

Thick biofilms (microbial mats) dominated by cyanobacteria occur in marine and freshwater habitats, and are especially well developed in extreme aquatic environments, including in the Arctic^1^ and Antarctica.^2^ These biofilms have a complex structural organization that facilitates nutrient recycling,^3^ and they often dominate ecosystem biomass^2^ and biological production^1, 4^ in the shallow lakes and ponds that are a major feature of the Arctic landscape.^5^ Although many of these high latitude water bodies freeze to the bottom during winter darkness, recent climate warming has led to reductions in bedfast ice,^6^ allowing microbial communities to persist in liquid water throughout the year. Our aim in the present study was to evaluate how this transition from seasonally frozen to perennially unfrozen conditions might affect the microbiome structure of Arctic biofilms, and the associated potential for biogeochemical processes.

We focused our sampling on Ward Hunt Lake (WHL) that harbors both seasonally frozen and perennially unfrozen microbial biofilms. WHL is the most northern lake in Canada, and is located in the northern Ellesmere Island region, 770 km from the North Pole (Fig. 1a). Previous studies^1, 7, 8^ at WHL have focused on benthic biofilms in the shallow moat of open water that forms in summer (Fig. 1b) and that refreezes to the lake sediment in early winter. It was assumed that benthic communities were restricted to this moat area because earlier ice coring in the central part of the lake had recorded 3.5–4.3 m of ice that extended to the lake bed. However, ground penetrating radar surveys^9^ revealed areas in the lake with up to 8 m of water below the maximum recorded ice thickness. In 2014, an extensive benthic biofilm was discovered in this deep, perennially ice-covered zone of WHL (Supplementary Video 1), and was sampled the following year.

**Fig. 1:**
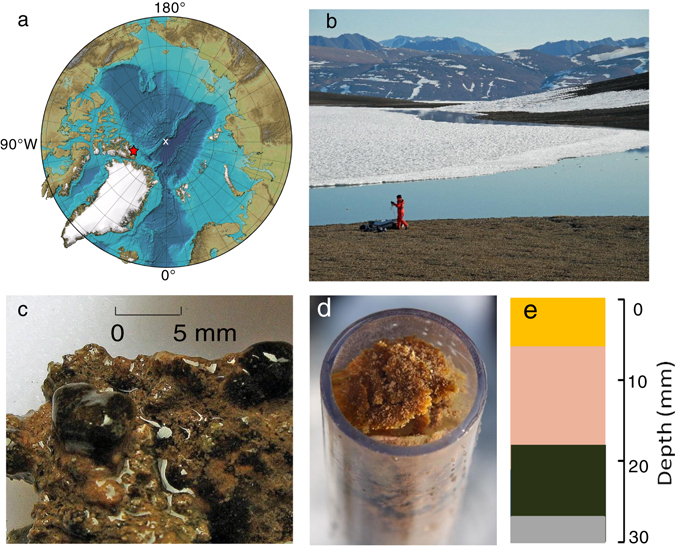
The high Arctic sampling site and biofilms. **a** Location of Ward Hunt Lake (*red star*) relative to the North Pole (*white cross*); the base map is the International Bathymetric Chart of the Arctic Ocean (IBCAO) of the Intergovernmental Oceanographic Commission (IOC), version 3.0 released in the public domain on 8 June 2012;^20^ **b** Ward Hunt Lake in mid-July 2015 showing the littoral open water zone (moat) and multi-year ice over the deeper waters of the lake; **c** Shallow biofilm over the rocks in the moat zone; **d** Sample of the deep biofilm in a mini-Glew sediment core (38 mm diameter) from 10 m depth; and **e** Schematic diagram of the biofilm color zonation (description in Supplementary Results)

We hypothesized that the deep biofilm communities of WHL would have a lower alpha-diversity than the moat biofilms because they would be dominated by microbial specialists that were adapted to a low solar energy regime caused by the thick perennial ice, combined with low nutrient conditions in this oligotrophic lake. In contrast, the moat habitat is exposed to full sunlight in summer, and receives direct nutrient subsidies via water tracks that discharge into it from the permafrost catchment; these warm (up to 8.8 °C) inflows may be mostly restricted to the moat zone and outflow, while the offshore waters are inversely stratified and typically <4 °C.^9^. To test this hypothesis of contrasting diversity, we analyzed three samples from each lake region (deep and moat microbial biofilms). DNA and RNA were extracted from the upper 3 to 5 mm (Fig. 1c–e) of the mats for high-throughput Illumina MiSeq amplicon sequencing that targeted variable regions of the small subunit ribosomal RNA (rRNA from complementary DNA (cDNA)) and the SS rRNA genes (rDNA from DNA). Detailed methods for collection, extraction, domain specific primers, and amplification steps are provided in the Supplementary Methods online. Raw reads were quality controlled and clustered into operational taxonomic units (OTUs) (Supplementary Table S1) with the UPARSE pipeline^10^ and these were further analyzed in QIIME^11^ (Supplementary methods). Raw bacterial, archaeal, and microbial eukaryote reads are available from the NCBI sequence read archive (SRA accession SRP078933).

This analysis of the WHL deep microbiome revealed a phylogenetic composition that differed markedly from the moat biofilms (Fig. 2). We found a high proportion of active OTUs in both communities, with the rRNA OTUs accounting for 72–88 % of the rDNA OTUs (Supplementary Table S2). Results from the phylogenetic clustering (Fig. 2 and Supplementary Fig. S1) and net relatedness indices (Supplementary Fig. S2) were consistent with habitat filtering^12^ (environmental selection of specific genotypes). Taxon abundances that differed by habitat (Fig. 2) included pennate diatoms, which were the predominant eukaryotes in the deep biofilms. Diatoms also tend to be abundant in Antarctic lake mats found under perennial ice cover^13, 14^ and are known to tolerate a range of light conditions, with a potential for heterotrophic growth when light becomes limiting.^15^ In contrast, heterotrophic ciliates were the dominant microbial eukaryotes in the moat mats, suggesting a more active microbial food web and dominance of heterotrophy, with ciliates grazing on other eukaryotes and bacteria.^16, 17^

**Fig. 2:**
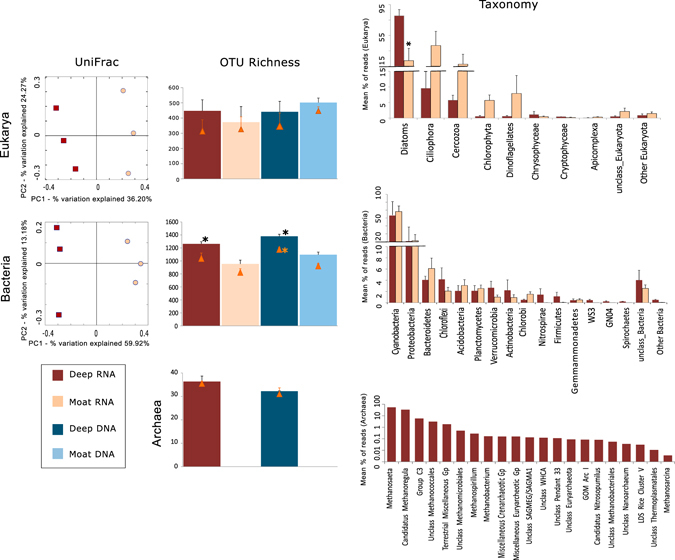
Comparison of microbiome structure in the deep and moat biofilms in Ward Hunt Lake. *Left panel*: PCoA plots showing the clustering of eukaryotic and bacterial rRNA samples according to the unweighted UniFrac metrics. *Middle panel*: Bar chart showing the Chao1 richness indices with standard error bars for triplicate rRNA and rDNA samples. Observed OTUs (*filled triangles*) with standard error bars are also indicated. *Right panel*: Taxonomic distribution for Eukarya (major groups), Bacteria (phyla) and Archaea (genera). Standard error bars are also shown. *Asterisks* indicate significant differences between deep vs. moat samples (*t*-test, *p* < 0.05). The PCoA and taxonomic results shown are for the rRNA analysis; rDNA results are given in Supplementary Fig. S1

Cyanobacteria were the most abundant bacteria in both deep and moat communities in WHL (Fig. 2), but differences in the cyanobacterial assemblages were observed between the two habitats (Supplementary Fig. S3). *Leptolyngbya* (Supplementary Fig. S3) was the most abundant cyanobacterial genus, accounting for 64–65% of cyanobacterial RNA reads in both communities. *Leptolyngbya* is a filamentous, globally distributed genus found in tropical,^18^ temperate,^19^ and polar microbial mats.^4^ At a finer taxonomic scale, many of the abundant OTUs were found to be closely related to Antarctic cyanobacteria (Supplementary Fig. S4), consistent with the global distribution of cold-dwelling ecotypes of cyanobacteria.^7^

Similar to Antarctic lake communities,^4^ potentially nitrogen-fixing cyanobacteria of the order *Nostocales* were rare in the deep biofilms (<0.01 % of bacterial reads) but abundant in the moat communities (6.2 %; Supplementary Fig. S3), that might indicate N-limitation or greater relative phosphorus availability in the moat area. As a result of this taxonomic distribution, the PICRUSt analysis of the cDNA reads predicted a greater percentage of the nitrogenase (*nifH*) gene in the moat microbiome (Supplementary Table S3).

Eukaryote and bacteria OTU Chao1 richness estimates for the deep mat microbiome were high relative to the moat biofilms (Fig. 2). Compared to the moat biofilms, there were more bacterial taxa normally associated with anaerobic conditions in the deep biofilms, including sulfate-reducing delta-proteobacteria, Chloroflexi, WS3, GN04, Spirochaetes, *Clostridia*, denitrifying genus *Thiobacillus* (Supplementary Fig. S5) and anaerobic methanogenic Archaea. By association, these and other taxa in the deep mats would have genes involved in denitrification and sulfate-reduction (PICRUSt results; Supplementary Table S3). Archaea were rare in the moat mats (Supplementary Results) and the presence of groups involved in methane cycling (e.g., *Methanomicrobia;* Fig. 2) along with methanotrophic bacteria (Supplementary Results) in the deep biofilms suggest a much larger potential for methane cycling in the deep communities.

In summer, the deep communities were in contact with well oxygenated water at the time of sampling, however, oxygen profiles indicated near-zero oxygen concentrations (2.9% of air equilibrium) overlying the biofilms in spring (Supplementary Fig. S6), and anoxia is, therefore, likely during winter darkness. Microzones of anoxia could persist in the biofilms during summer, as reported in temperate systems.^19^ Oxygen availability has been shown to have a structuring effect on mat communities in Antarctic ponds,^4^ and the seasonal fluctuations in both bottom water and biofilm oxygen may operate similarly in WHL, ensuring the persistence of anaerobic taxa in the deep biofilms through much of the year while aerobic taxa become active in summer. In contrast to the deep zone of the lake that does not freeze to the bottom in winter, the water column of the moat freezes solid each year and there is no liquid water during winter darkness to allow such alternation of oxic and anoxic niches for microbial activation and growth. There is presently no summer bedfast ice in the lake, and, therefore, no benthic sites that remain frozen throughout the year.

Large changes in ice cover have already been observed throughout the Arctic, with fewer lakes freezing to the bottom as the thickness of winter ice decreases.^6^ WHL lost its ice cover on two occasions in recent years,^9^ and a shift from multi-year ice to full ice-out conditions in summer could expand the habitat for communities that remain active during winter. The increased prevalence of liquid water conditions during winter darkness will likely favor anaerobic processes in the benthic microbiomes of Arctic lakes, and the resultant increases in methane production and denitrification will need to be accounted for in global carbon and nitrogen budgets. All of these aspects will require additional studies throughout the circumpolar North to determine whether benthic anoxia is a general feature of lakes without bedfast ice during the winter dark season, and to evaluate whether these environments support a diverse biofilm community of aerobes and anaerobes, as observed here at WHL.

## Data availability

Sequence data have been deposited in the NCBI sequence read archive (SRA accession SRP078933). The environmental data are available in the Nordicana D archive (doi: 10.5885/45445CE-7B8194).

## Electronic supplementary material

Supplemental Material Guide

Supplemental Information

Video 1
