# Supplementary material for: Hidden biofilms in a far northern lake and implications for the changing Arctic
Source: NPJ Biofilms Microbiomes. 2017 Jul 6;3:17. doi: 10.1038/s41522-017-0024-3 (PMC5500582; doi:10.1038/s41522-017-0024-3)
Supplement: Supplementary file 1 — Supplemental Material Guide [file 41522_2017_24_MOESM1_ESM.doc]

**Hidden biofilms in a far northern lake and implications for the changing Arctic**

V. Mohit, A. Culley, C. Lovejoy, F. Bouchard and W. F. Vincent

**SI Guide**

1. Supplementary Information file

This contains the following supplementary text and 9 display items: Supplementary Materials and Methods; Supplementary Results; Supplementary References; Supplementary Tables (Supplementary Tables S1 – S3); and Supplementary Figures (Supplementary Figures S1 – S6);

2. Supplementary video file

Video 1. Underwater video at the deep site of Ward Hunt Lake in the Canadian High Arctic, July 2014. The video camera was lowered through a hole in the 2-m ice cover then down the 8-m water column beneath, to the sediments overlaid with microbial biofilms and sporadic patches of biofilm-coated moss.
