# Supplementary material for: Hidden biofilms in a far northern lake and implications for the changing Arctic
Source: NPJ Biofilms Microbiomes. 2017 Jul 6;3:17. doi: 10.1038/s41522-017-0024-3 (PMC5500582; doi:10.1038/s41522-017-0024-3)
Supplement: Supplementary file 2 — Supplemental Information [file 41522_2017_24_MOESM2_ESM.pdf]

Hidden biofilms in a far northern lake and implications for the changing Arctic

V. Mohit, A. Culley, C. Lovejoy, F. Bouchard, and W. F. Vincent

Supplementary Information Content:

Supplementary Materials and Methods

Supplementary Results

Supplementary References

Supplementary Tables (Supplementary Tables S1 – S3)

Supplementary Figures (Supplementary Figures S1 – S6)

Supplementary Materials and Methods:

Study Site

Ward Hunt Lake (WHL) (lat. 83°05'N, long. 74°10'W) is located on Ward Hunt Island, 6 km off the northern coast of Ellesmere Island, in the northernmost region of High Arctic Canada (Figure 1a). The lake has a polar desert catchment with mean annual air temperatures around -17°C, similar to analogous regions of perennially ice-capped lakes in Antarctica, but with higher precipitation (around 150 mm) [1,2]. WHL has an area of 0.37 km^2 and a maximum depth[3] of 10 m. WHL is fully ice-covered for 10 months of the year, and mid-summer ice thickness can be up to 4 m. In late summer, a moat of open water, up to 15 m wide, forms in the littoral zone (Figure 1b). Since 2008, there has been a trend of decreasing ice thickness and the lake was completely ice-free in summers of 2011 and 2012. Between 2013 and 2016, the midsummer ice thickness [3] has averaged approximately 2 m. WHL is considered oligotrophic [4], with low concentrations of nutrients; e.g., soluble reactive phosphorus <0.5 µg L^{-1} and chlorophyll *a* <1.0 µg L^{-1}. However, high nutrient concentrations are recorded for the interstitial fluids in microbial biofilms from the littoral zones that are 2.5 to 43 times higher than those of the overlying water column.[4] WHL has a simplified planktonic food web with no observed macro-zooplankton or fish populations, however chironomid tubes occur over the sediments in shallow waters. The lake is fed by accumulated snow that melts and discharges via water tracks that run over the permafrost table. [3]

Biofilm observations and sampling

In July 2014, a GoPro HERO3 video camera in a waterproof housing was lowered through holes drilled through the ice over the deepest part of the lake (Supplementary Video 1) and provided the first indications of benthic biofilms (microbial mat communities). The first samples of this deep community (10 m depth relative to the upper ice surface; lat. 83°N 05.242; long. 74°W 08.862) were collected the next summer (July 2015) using a 38-mm mini-Glew gravity corer [5] lowered through 20-cm diameter holes drilled in the ice at three replicate sites spaced 5 m apart. Previous studies of polar microbial biofilms (Antarctic cyanobacterial mats) have indicated that three replicates would be of sufficient power to detect depth effects [2], and our subsequent analyses showed that the triplicates were well clustered relative to the differences between depth zones. At the time of sampling, there was no snow on top of the perennial ice and the ice thickness was 2.1 m. The lake ice consisted of a surface 5 cm layer of white ice underlain by clear congelation ice. The sediment cores were extruded though the coring barrel and the surface 2-3 mm removed with a spatula. During the same week, shallow biofilms in the ice-free inshore open-water (moat) area of the littoral zone were collected at lat. 83°N 05.295, long. 74°W 09.763 from 30 cm depth with sterile gloves, at three replicate sites 5 m apart. The 2 - 4 mm-thick biofilms were peeled off their rocky substrate and sampled in their entirety. The two types of microbial samples are referred to as deep and moat biofilms, respectively, in the text. The biofilms were placed immediately in RNAlater (Ambion, Texas, USA), stored in the field at -20°C in a solar powered freezer, and then transferred to -80°C until nucleic acid extraction within one month of sample collection. Oxygen measurements were made with a Yellow Springs Instrument submersible profiler

(YSI, Yellow Springs, Ohio, USA). Irradiance was measured with an underwater quantum sensor LI-192 (LI-COR, Nebraska, USA). All limnological data from this study are available in the online repository Nordicana D. [6]

Nucleic acid extraction, amplification and sequencing

The biofilm samples were rinsed three times with phosphate buffer saline solution and the RNA and DNA then extracted using the RNA PowerSoil Total RNA Isolation and DNA Elution Accessory kits (MO BIO Laboratories Inc., Carlsbad, CA, USA), according to the manufacturer's protocol. Eluted RNA was treated with TURBO DNase (Ambion, Texas, USA) and converted to complementary DNA (cDNA) using the High Capacity cDNA Reverse Transcription kit (Applied Biosystems, CA, USA). The V4 (eukaryotes), V6-V8 (Bacteria) and V3-V5 (Archaea) regions of the SS rRNA and the SS rRNA gene were amplified using the primers described in Comeau et al. [7], but with Illumina adaptors, and an annealing temperature for the Archaea-targeted PCR reactions of 52°C. The amplicons were verified by running the PCR products on 1% agarose gels in Tris-Borate-EDTA buffer, purified using Axygen magnetic beads (Corning Life Sciences, NY, USA) and quantified spectrophotometrically with a Nanodrop ND 1000. The samples were diluted 10 – 50X before performing a second round of PCR on the purified products, with the same adapter sequence as in the first PCR. The protocol for this second PCR was: initial denaturation at 98°C for 30 s, 13 cycles of denaturation at 98°C for 10 s, annealing at 55°C for 30 s, elongation at 72°C for 30 s and a final elongation step at 72° C for 4.5 min. A second bead-purification step was performed

before spectrophotometric quantification. Amplicons were pooled equimolarly and paired-end sequencing was performed on a MiSeq Illumina system at the Institut de Biologie Intégrative et des Systèmes (IBIS), Université Laval Plateforme d'Analyses Génomiques, Québec, Canada. The raw reads have been deposited in the NCBI Sequence Read Archive (SRA; http://www.ncbi.nlm.nih.gov/sra) under the accession number SRP078933.

Sequence analysis

The paired-end reads were merged and then preliminary quality control of the Illumina reads was performed with the UPARSE [8] pipeline implemented in USEARCH (http://drive5.com/usearch/manual/upp_ill_pe.html) to discard reads with low Q scores, short reads (< 300 nt) and singletons. The datasets for each microbial domain were subsampled to 20100 (eukaryotes), 39800 (Bacteria) or 55500 (Archaea) reads for comparative analyses among samples. The resulting reads were clustered into operational taxonomic units (OTUs), using a 98% sequence identity cut-off for eukaryotic and a 97% cut-off for bacterial and archaeal reads. Chimeras were identified and removed via the UCHIME algorithm [9] implemented in USEARCH through a comparison of our dataset with the SILVA 18S and 16S rRNA sequence databases. Representative OTUs were classified with mothur [10] based on searches of custom-curated eukaryotic, bacterial and archaeal databases [11]. Representative OTU sequences were aligned with NAST [12] against the SILVA 18S and 16S rRNA datasets. Non-aligned sequences and also those corresponding to fungi, metazoa and streptophytes were removed from the eukaryotic dataset after the taxonomic classification step. The taxonomy of the dominant unclassified eukaryotic OTUs (>1% of the total reads in the whole dataset) was further

investigated by the Evolutionary Placement Algorithm (EPA) based on a reference phylogenetic tree built with BLASTn (http://blast.ncbi.nlm.nih.gov/) hits. Remaining non-microbial eukaryotic reads were removed from the dataset. Construction of the reference tree and the EPA placement of the eukaryotic reads were done with the Randomized Axelerated Maximum Likelihood (RAxML) v8.2.0 [13] platform. Representative OTUs in the bacterial and archaeal dataset were also classified with the RDP classifier [14]; unrooted sequences and those classified as Archaea in RDP as well as chloroplast sequences were eliminated from the bacterial dataset. Given that Cyanobacteria are key phototrophic taxa in Arctic microbial biofilms [15,16], the taxonomy of the top 12 most dominant cyanobacterial OTUs were further investigated with EPA. The taxonomy of archaeal OTUs was also refined with EPA placement on a reference tree. A summary of the number of raw reads, final quality controlled reads, exclusivity to the respective domains and the number of OTUs observed in each sample is given in Table S1. The archaeal reads from the moat biofilms were removed from further statistical analysis given the low number (17–3127) of high-quality archaeal reads obtained from these samples.

Alpha-diversity

QIIME 1.6.0 [17] was used for calculations of alpha-diversity (Chao1 richness estimates and observed OTUs) and beta-diversity. The net relatedness index (NRI), an index for community phylogenetic structure [18] was calculated for each sample, using the picante package in R v3.2.3 [19]. NRI measures the degree of phylogenetic clustering of taxa in each sample relative to the pool of taxa in the whole dataset across a phylogenetic tree. Separate phylogenetic trees were constructed with FastTree

(www.microbesonline.org/fasttree/) for RNA and DNA samples. Abundance weighted mean pair-wise distances (MPD) were calculated against a null 'taxa.labels' model of 999 randomized trees. The generated standardized metric (SES_{mpd}) corresponded to negative 1 x NRI. The PAST statistics software package (http://folk.uio.no/ohammer/past/) was used to calculate the Menhinick richness index for OTUs within a subsample of anaerobic taxa; this index is independent of sample size.

Beta-diversity

Weighted and unweighted jackknifed UniFrac distance metrics [20] were calculated in QIIME for both the RNA (Figure 2) and DNA datasets (Figure S1). Sample clustering was visualized as Principal coordinates analysis (PCoA) plots based on the UniFrac distance (Figure 2, Figure S1). Morisita-Horn distance was used to evaluate the clustering of samples based on cyanobacterial taxa. The Morisita-Horn metrics were used because of its lower sensitivity to sample size [21,22] to perform the cyanobacterial-specific clustering analysis on non-rarefied datasets. UniFrac clustering analysis was also performed on the Cyanobacteria-only datasets by rarefying the rRNA dataset to 7000 reads and the rDNA dataset to 2000 reads.

Metagenome prediction with PICRUSt

PICRUSt [23] was used to predict the microbial community metagenome based on 16S rRNA, to infer some of the functional potential of the active bacterial communities (16S rRNA) in the microbiomes. OTUs were selected and annotated against the Greengenes 13_5 reference database using the pick_closed_reference_otus.py script in QIIME. The resulting OTU table was used as input in PICRUSt, which was accessed via

the Galaxy interface (huttenhower.sph.harvard.edu/galaxy/). The potential gene abundance in the OTU table was normalized by 16S rRNA copy number.

Statistical analysis

Statistical analyses were performed with the PAST statistics software package. The Shapiro-Wilk test was used to verify normality of distribution, and F-tests used to compare the variances between samples. Significant differences in alpha-diversity metrics, PICRUSt gene abundances and in taxonomic distribution were assessed via t-tests; normality and equality of variance were tested and confirmed in all cases (F-test at the 0.05 significance level).

Supplementary Results

Microbial biofilms: Stratigraphy and morphological characteristics

The underwater observations at the deep water site (Supplementary video 1) revealed that the microbial community formed a mostly continuous biofilm over the lake floor at the deep-water location, with sporadic patches of aquatic mosses that were also coated with biofilms. The sediment cores were composed of a 3-5 mm orange-brown, loosely cohesive layer overlying a 10-15 mm thick beige coloured zone of sediment. This overlaid a 5-10 mm zone of olive-black sediment with thin (0.2 mm) membranous layers, in turn underlain by a light grey substrate (Figure 1d, 1e). Visually the moat biofilms (depth = 0.1 - 0.5 m) were patchily distributed and were composed of a cohesive 3 - 5

mm pink layer that was intercalated with green layers and dotted with black colonies at the surface, with a thin blue-green under-surface overlying the bottom substrate of gravel and sand. Further details of the moat biofilms are given in Villeneuve *et al.* [4], Lionard *et al.* [15] and Jungblut *et al*. [16]

Light and oxygen conditions

Midday downwelling irradiance at the time of sampling was 2.5 % of incident at 10 m at the deep site and 55.4% of incident at 0.3 m in the moat, at the sampling site shown in Figure 1b. Oxygen in the moat zone was 109% of air-equilibrium (12.6 mg O_2 L^{-1}, 5.8°C), and at 81% (10.1 mg O_2 L^{-1}, 5.1°C) above the deep biofilms at the times of sampling. Subsequent measurements the next year (2016 field season) showed an oxygen saturation of 21.1% (2.8 mg O_2 L^{-1}) above the deep biofilms in July (mid-summer), but near anoxic conditions (at the detection limit of the YSI probe: 2.9 % oxygen saturation, 0.4 mg O_2 L^{-1}) in spring in May (Figure S6).

Alpha-diversity

Bacterial Chao1 richness were significantly greater (t-test, $p<0.05$) in the deep biofilms compared to the moat communities for both the rRNA (Figure 2) and rDNA sourced communities. Observed OTUs were significantly greater in the deep than the moat mats only for the bacterial rDNA samples. Menhinick richness index showed a higher OTU richness in the deep (rRNA: 2.7, rDNA: 1.7) than in the moat mats (rRNA: 2.7, rDNA: 1.2) for the identified anaerobic taxa. The higher richness suggests higher functional diversity in the deep biofilms, including possibly in terms of anaerobic processes, and the higher Chao1 richness estimate in the deep mat bacterial communities

could be due to greater abundance of rare OTUs in those mats. Further analysis of the deep biofilm sequences with PICRUSt predicted a significantly higher abundance ($p<0.05$) of the nitrite reductase (*nirS*) and higher abundance of sulphite reductase (*DSR*) genes involved in denitrification and sulphate reduction respectively (Table S3).

The positive net relatedness indices (NRI) (Figure S2) and the phylogenetic clustering of the deep versus moat communities according to the UniFrac [7] unweighted (Figure 2) and weighted metrics (not shown for rRNA fraction) both at the rDNA (Figure S1) and rRNA level indicate that habitat type was a determining factor in the structure of the active microbial assemblages. A large proportion of the biofilm constituents from all three microbial domains were potentially active. A comparison of the shared number of OTUs present in the total community at the rDNA level versus the potentially active community at the rRNA level, revealed that a high proportion of reads (72 – 88%) were shared between the two fractions (Table S2).

Bacterial distribution

Cyanobacteria were the most abundant group of bacteria in the surface layer of both types of biofilms (Figure 2). The second most abundant group, were bacteria from the phylum *Proteobacteria,* and were composed of strictly anaerobic sulphate-reducing deltaproteobacteria. These were from the orders *Syntrophobacterales*, *Desulfobacterales* and *Desulfuromonadales,* and were in higher abundance in the deep biofilms (totalling 22.2 % of the active *Deltaproteobacteria*) than in the moat (0.7 %) biofilms (Figure S5). *Chloroflexi*, mainly composed of bacteria in the class *Anaerolineae*, was also among the most abundant groups in both communities (Figure 2, Figure S5). Bacteria in the

Anaerolineae are mainly anaerobic and are involved in carbon degradation .[24] Other strict anaerobes in the class *Clostridia* were only present and active (1.1% of rRNA reads) in the deep communities (Figure S5). Other active anaerobic taxa from the phyla WS3 and Spirochaetes were significantly higher in the deep mats than in the moat mats (Figure S5, t-test, $p<0.05$). The denitrifying bacterial genus *Thiobacillus* was more abundant in the active deep (0.41 %) than in the moat (0.002 %) microbial mat communities. With respect to the rDNA samples, WS3, the anaerobic sulfate reducing deltaproteobacteria and *Thiobacillus* were either significantly lower or absent in the moat than in the deep mats (Figure S5). Cumulatively, these data suggest a higher incidence of anaerobic niches in the deep biofilm.

Methanotrophic bacteria from the family *Methylococcaceae* were recovered in the deep biofilms (0.14 %), but were rare in the moat communities (< 0.01 %), an indication that methane is more available in the deep water habitat and derived from methanogenesis under anaerobic conditions. Nitrifiers in the genera *Nitrosospira* (0.03 %) and *Nitrospira* (1.4 %) were lower in abundance in the moat communities (<0.01 % and 0.2%, respectively) and this can also suggest that higher functional diversity pertains in the deep biofilms. Reads for rRNA belonging to the nitrifying archaeal group *Nitrosopumilus* (Phylum *Thaumarchaeota*) [25] were also recovered from the deep biofilms (Figure 2).

Cyanobacterial diversity and composition

Both the Morisita-Horn and Unifrac clustering analysis in the rRNA and rDNA fractions showed distinct clustering of the moat cyanobacterial communities from the

deep ones (Figures S3A and B). In both analyses, the replicate moat communities clustered closer to each other than the replicate deep mat cyanobacterial communities, where one replicate was further apart. The rDNA UniFrac analysis (not shown) showed a similar clustering pattern as that of the rRNA analysis (Figure S3B). This suggests that the cyanobacterial communities contribute to the distinct bacterial assemblages observed in the deep versus the moat mats.

The most abundant OTU in the deep community (OTU2), with a mean relative abundance of 40.4 % of active (rRNA) bacteria, was identical to *Leptolyngbya* sp. CYN68 (Figure S3), a filamentous cyanobacterium isolated from a pond in Antarctica .[26] The most abundant cyanobacterial OTU in the moat communities (OTU 8), with a mean relative abundance of 11.0 % of active bacteria, was homologous to the *Leptolyngbya* sp. CENA375 and *Phormidium* sp. SAG 37.90 clusters according to EPA analysis (Figure S3). *Phormidium* sp. SAG 37.90 was also closely related to an OTU identified in a previous study of Ward Hunt Lake moat communties[16]. OTU1228, OTU9 and OTU3 clustered with Antarctic cyanobacterial isolates (Figure S3), consistent with the existence of cold-habitat-specific ecotypes [16]. There were marked differences between the percentage abundances of the dominant cyanobacterial OTUs between the two types of biofilms (Figure S3), which together with the Morisita-Horn results, implies that habitat filtering affected cyanobacterial community structure.

Archaea in the moat community

Archaeal reads from the moat biofilm samples were not analysed quantitatively because of the low recovery of high quality archaeal-related reads (Table S3). More PCR optimisation was needed to obtain amplicons from the moat mats. Also, 12 – 23 % of the

rDNA and 26 – 28 % of rRNA raw reads in the archaeal datasets were removed because they did not align well to the rest of the reads. These unaligned reads corresponded mainly to bacterial or unrooted (neither Bacteria or Archaea) taxa. This suggests that archaeal templates were in low abundance in this littoral community. Metagenomic profiling of annually frozen Arctic and Antarctic biofilms similar to those in Ward Hunt Lake also showed a minimal contribution of Archaea to the overall communities.[27]

Supplementary References

1 Vincent WF, Fortier D, Lévesque E, Boulanger-Lapointe N, Tremblay B, Sarrazin D *et al.* Extreme ecosystems and geosystems in the Canadian High Arctic: Ward Hunt Island and vicinity. *Ecoscience* **18**: 236–261 (2011)

2 Jungblut AD, Hawes I, Mackey TJ, Krusor M, Doran PT, Sumner DY *et al.* Microbial mat communities along an oxygen gradient in a perennially ice-covered Antarctic lake. *Appl Environ Microbiol* **82**: 620–630 (2016)

3 Paquette M, Fortier D, Mueller DR, Sarrazin D, Vincent WF. Rapid disappearance of perennial ice on Canada's most northern lake. *Geophys Res Lett* **42**: 1433–1440 (2015)

4 Villeneuve V, Vincent WF, Komarek J. Community structure and microhabitat characteristics of cyanobacterial mats in an extreme High Arctic environment: Ward Hunt Lake. *Nov Hedwigia* **123**: 199–224 (2001)

5 Glew JR. Miniature gravity corer for recovering short sediment cores. *J Paleolimnol* **5**: 285–287 (1991)

6 NEIGE. Water column physico-chemical profiles of lakes and fiords along the northern coastline of Ellesmere Island, v. 1.1 (1954-2016). *Nordicana* **D27**, doi: 10.5885/45445CE-7B8194 (2016)

7 Comeau AM, Li WKW, Tremblay J-É, Carmack EC, Lovejoy C. Arctic Ocean microbial community structure before and after the 2007 record sea ice minimum. *PLoS One* **6**: e27492 (2011)

8 Edgar RC. UPARSE: highly accurate OTU sequences from microbial amplicon reads. *Nat Methods* **10**: 996–8 (2013)

9 Edgar RC, Haas BJ, Clemente JC, Quince C, Knight R. UCHIME improves sensitivity and speed of chimera detection. *Bioinformatics* **27**: 2194–200 (2011)

10 Schloss PD, Westcott SL, Ryabin T, Hall JR, Hartmann M, Hollister EB *et al.* Introducing mothur: open-source, platform-independent, community-supported software for describing and comparing microbial communities. *Appl Environ Microbiol* **75**: 7537–41 (2009)

11 Lovejoy C, Comeau AM, Thaler M. Curated reference database of SSU rRNA for northern marine and freshwater communities of Archaea, Bacteria and microbial eukaryotes. *Nordicana* **D23**, v. 1.1 (2002-2008), doi:10.5885/45409XD-79A199B76BCC4110 (2016)

12 Caporaso JG, Bittinger K, Bushman FD, Desantis TZ, Andersen GL, Knight R. PyNAST: A flexible tool for aligning sequences to a template alignment. *Bioinformatics* **26**: 266–267 (2010)

13 Stamatakis A. RAxML-VI-HPC: maximum likelihood-based phylogenetic analyses with thousands of taxa and mixed models. *Bioinformatics* **22**: 2688–90 (2006)

14 Cole JR, Wang Q, Cardenas E, Fish J, Chai B, Farris RJ *et al.* The Ribosomal Database Project: improved alignments and new tools for rRNA analysis. *Nucleic Acids Res* **37**: D141–D145 (2009)

15 Lionard M, Péquin B, Lovejoy C, Vincent WF. Benthic cyanobacterial mats in the high arctic: multi-layer structure and fluorescence responses to osmotic stress. *Front Microbiol* **3**: 140 (2012)

16 Jungblut AD, Lovejoy C, Vincent WF. Global distribution of cyanobacterial ecotypes in the cold biosphere. *ISME J* **4**: 191–202 (2010)

17 Caporaso JG, Kuczynski J, Stombaugh J, Bittinger K, Bushman FD, Costello EK *et al.* QIIME allows analysis of high- throughput community sequencing data. *Nat Methods* **7**: 335–336 (2010)

18 Webb CO, Ackerly DD, McPeek MA, Donoghue MJ. Phylogenies and community ecology. *Annu Rev Ecol Syst* **33**: 475–505 (2002)

19 Kembel SW, Cowan PD, Helmus MR, Cornwell WK, Morlon H, Ackerly DD *et al.* Picante: R tools for integrating phylogenies and ecology. *Bioinformatics* **26**: 1463–1464 (2010)

20 Lozupone C, Knight R. UniFrac: A new phylogenetic method for comparing microbial communities. *Appl Environ Microbiol* **71**: 8228–8235 (2005)

21 Wolda H. Similarity indices, sample size and diversity. *Oecologia* **50**: 296–302 (1981)

22 Chao A, Chazdon RL, Colwell RK, Shen TJ. Abundance-based similarity indices and their estimation when there are unseen species in samples. *Biometrics* **62**: 361–371 (2006)

23 Langille MGI, Zaneveld J, Caporaso JG, McDonald D, Knights D, Reyes JA *et al.*

Predictive functional profiling of microbial communities using 16S rRNA marker gene sequences. *Nat Biotechnol* **31**: 814–21 (2013)

24 Jorgensen SL, Hannisdal B, Lanzen A, Baumberger T, Flesland K, Fonseca R *et al.* Correlating microbial community profiles with geochemical data in highly stratified sediments from the Arctic Mid-Ocean Ridge. *Proc Natl Acad Sci USA* **109**: E2846–E2855 (2012)

25 Pester M, Schleper C, Wagner M. The Thaumarchaeota: An emerging view of their phylogeny and ecophysiology. *Curr Opin Microbiol* **14**: 300–306 (2011)

26 Martineau E, Wood SA, Miller MR, Jungblut AD, Hawes I, Webster-Brown J *et al.* Characterisation of Antarctic cyanobacteria and comparison with New Zealand strains. *Hydrobiologia* **711**: 139–154 (2013)

27 Varin T, Lovejoy C, Jungblut AD, Vincent WF, Corbeil, J. Metagenomic profiling of Arctic microbial mat communities as nutrient scavenging and recycling systems. *Limnol Oceanogr* **55:** 1901–1911 (2010)

Supplementary Tables (Supplementary Tables S1 – S3)

Table S1: Summary of the raw reads, merged reads, final high-quality reads and Operational Taxonomic Unit (OTU) statistics. Samples were taken in triplicate from the deep and moat sites.

Zone	Replicate	Nucleic Acid	Total raw read pairs	Merged Reads	Final QC reads	OTUs	Total raw read pairs	Merged Reads	Final QC reads	OTUs	Total raw read pairs	Merged Reads	Final QC reads	OTUs
			Eukaryote[1]				Bacteria[2]				Archaea[3]			
Moat	1	RNA	282404	263374	70542	198	307272	280256	83100	810	255026	207751	17	-
		DNA	260856	241625	46626	416	275151	262245	93004	917	292470	212053	3127	-
	2	RNA	230288	211212	38028	479	220441	209053	48451	929	221320	196214	449	-
		DNA	240857	221117	33744	501	199238	189146	53319	987	235102	182717	2409	-
	3	RNA	270385	255638	22331	302	271878	258489	59263	730	243165	217217	202	-
		DNA	255796	234248	30724	422	247283	235479	71865	863	312483	230407	572	-
Deep	1	RNA	316807	299690	179667	294	253226	242248	81104	986	207702	191052	67632	31
		DNA	285489	263600	133075	371	215450	205143	57347	1205	255689	234807	84567	31
	2	RNA	348657	329599	196280	208	331358	312977	44190	1201	243286	220117	70350	40
		DNA	312457	291115	163546	259	243657	231605	48812	1214	240118	215566	61643	32
	3	RNA	328911	308297	105689	451	342918	327124	113200	926	245036	225387	76445	37
		DNA	266308	248197	96350	479	274004	261163	77228	1170	266286	248618	89770	30

Each sample was rarefied to:

[1] 20100 reads

[2] 39800 reads

[3] 55500 reads

Table S2: Tables showing the percentage of shared OTUs between rDNA and rRNA fractions in each biofilm type as defined in the text. The normalized dataset was used to calculate the percentages.

	Deep		Moat	
	% rDNA reads	% rRNA reads	% rDNA reads	% rRNA reads
Eukaryota	75	79	73	79
Bacteria	82	88	82	80
Archaea	88	72	-	-

Table S3: PICRUSt analysis showing the predicted percentage abundance of nifH, Nir and DSR genes in the deep and moat biofilms and the t-test results ($p < 0.05$) to test the differences in gene abundance between the two sites.

**KO designation; gene; function**	**Deep**	**Moat**	**p value**
K02588; nitrogenase nifH; Nitrogen fixation	0.0081%	0.0121%	0.048
K00368; nitrite reductase Nir; Denitrification	0.005%	0.001%	0.001
K11180; sulfite reductase DSR; Sulfate reduction	0.0040%	0.0002%	0.061

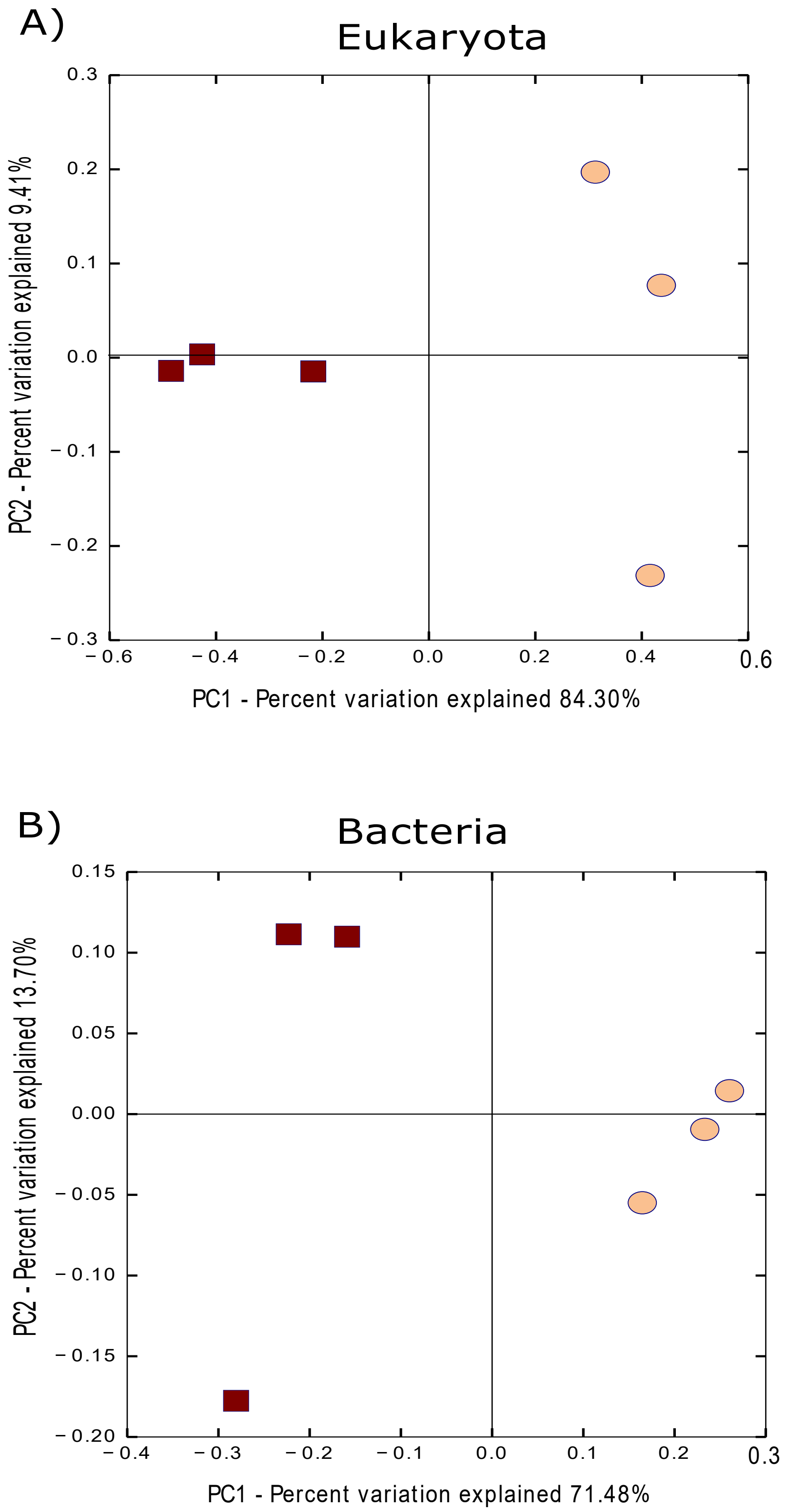

Figure S1: PCoA plots showing the clustering of eukaryotic (A) and bacterial (B) rDNA samples according to the weighted UniFrac metrics. Unweighted (Figure 2) and weighted clustering of the rRNA (not shown) samples showed similar patterns to the rDNA sample clustering.

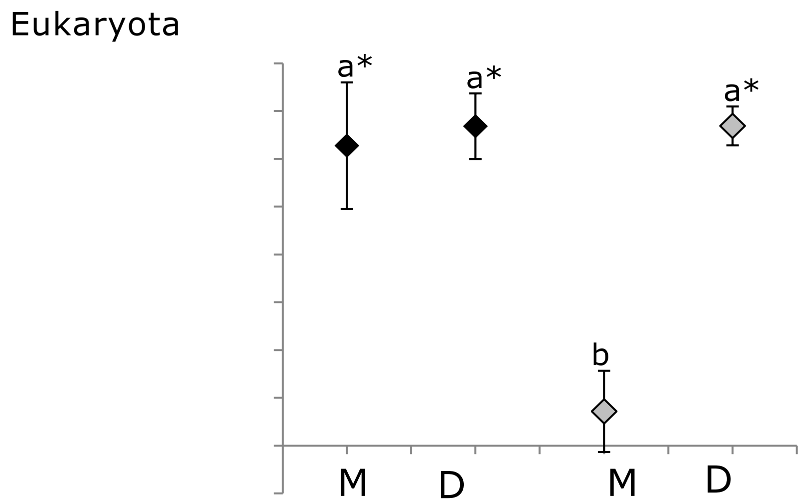

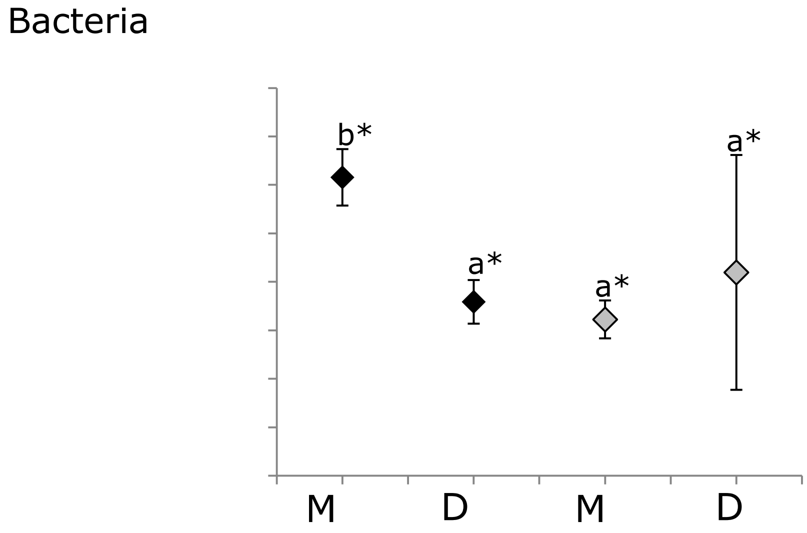

Figure S2: Mean weighted net relatedness indices (NRI) (±Standard Error) showing the phylogenetic clustering/dispersion of OTUs relative to RNA (black diamond) and DNA (grey diamond) sequence analysis. Asterisks indicate communities that are significantly structured ($p<0.05$ based on 999 random permutations of the taxa labels in the phylogenetic tree) in at least two replicate samples. Different letters indicate significantly different NRI values between the deep and moat biofilms (t-test, $p<0.05$). M: Moat; D: Deep.

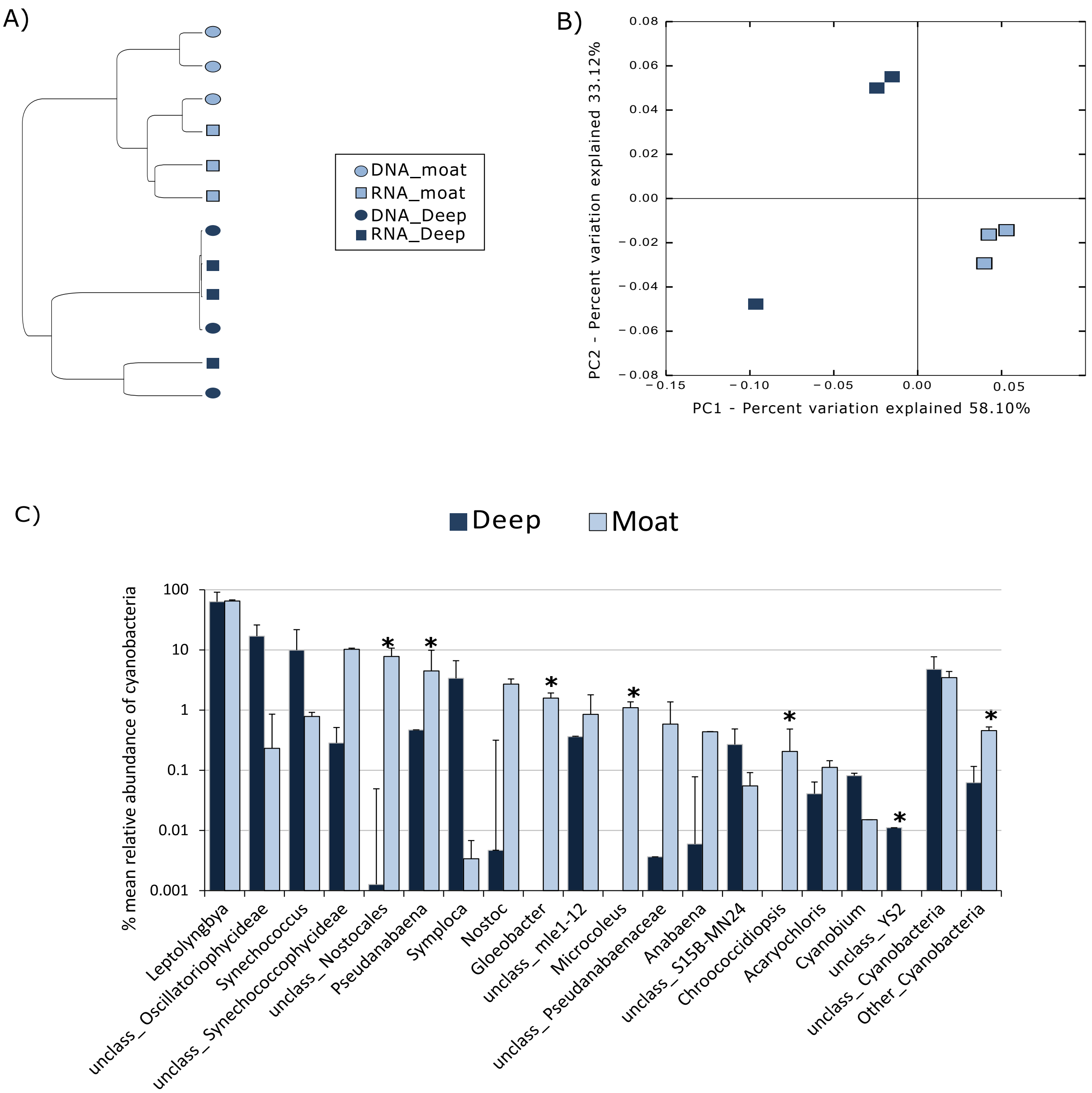

Figure S3: A) Morisita-Horn UPGMA tree of the sample clustering using the abundance of each OTU corresponding to cyanobacterial taxa, the dataset here was not rarefied. B) PCoA showing the weighted UniFrac clustering of the RNA cyanobacterial reads. C) Cyanobacterial distribution at the genus level. Note that the vertical axis is a log scale. Standard error bars are shown. Asterisks indicate significant difference between deep and moat samples (t-test, $p<0.05$).

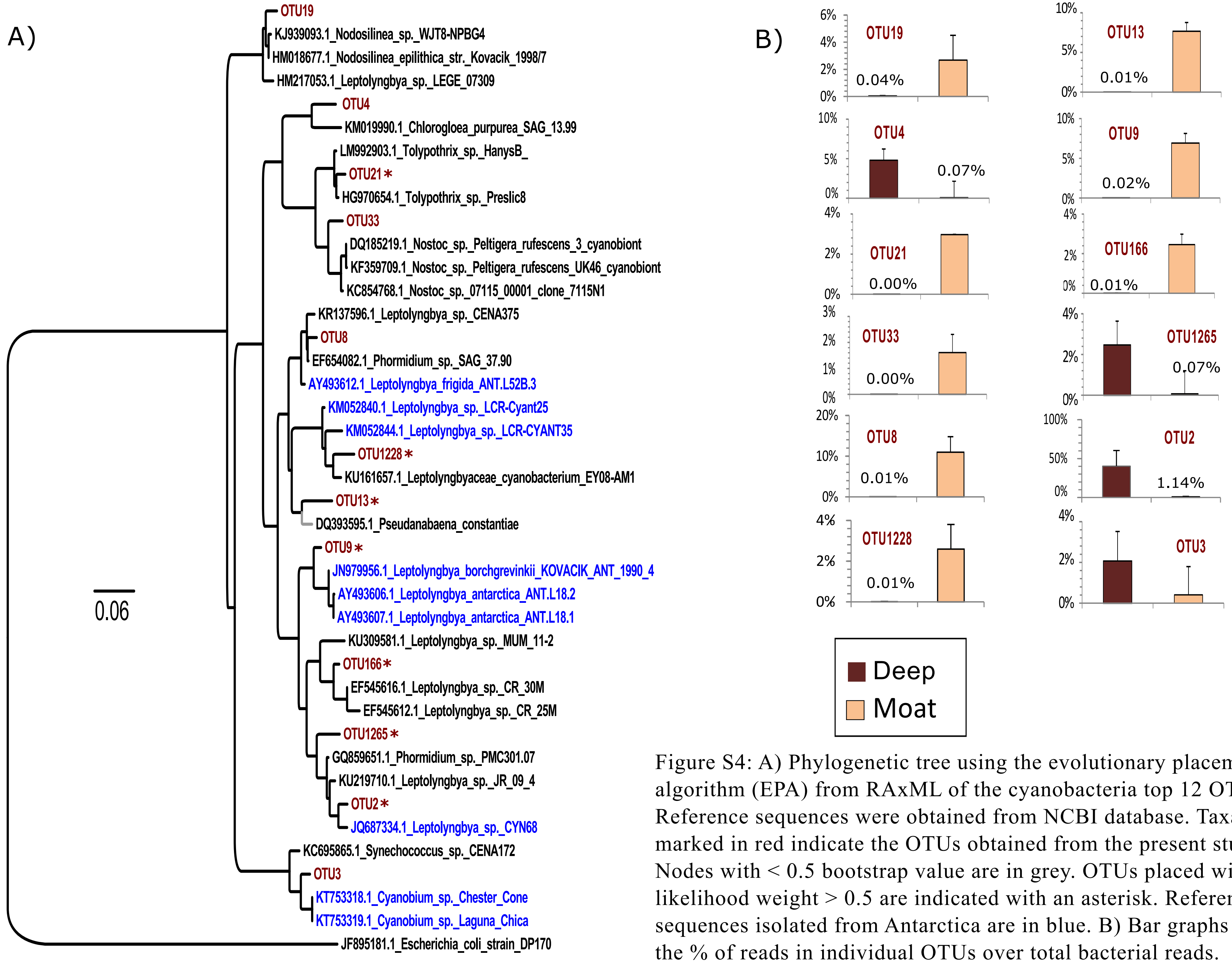

Figure S4: A) Phylogenetic tree using the evolutionary placement algorithm (EPA) from RAxML of the cyanobacteria top 12 OTUs. Reference sequences were obtained from NCBI database. Taxa marked in red indicate the OTUs obtained from the present study. Nodes with < 0.5 bootstrap value are in grey. OTUs placed with likelihood weight > 0.5 are indicated with an asterisk. Reference sequences isolated from Antarctica are in blue. B) Bar graphs show the % of reads in individual OTUs over total bacterial reads.

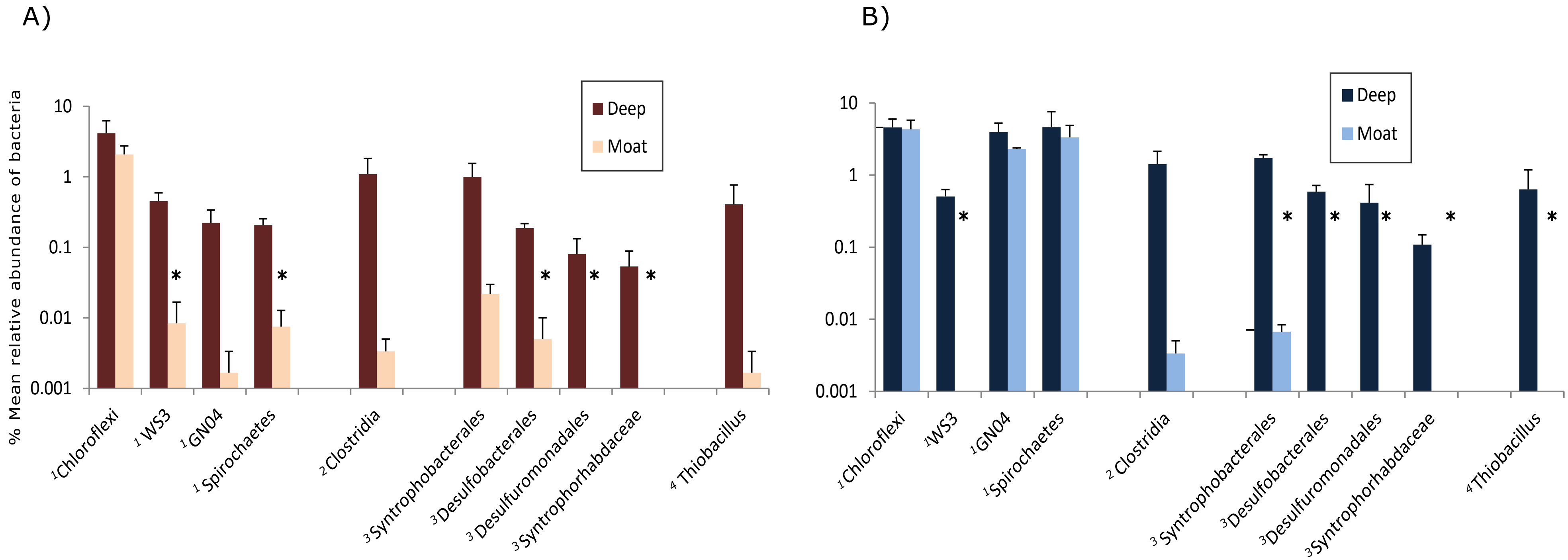

[1] Taxa classified at the phylum level
[2] Taxa classified at the class level
[3] Bacterial orders belonging to anaerobic sulfate reducing deltaproteobacteria
[4] Betaproteobacterial genus which participates in denitrification in anaerobic conditions

Figure S5: Percentage mean relative abundance of anaerobic taxa present in the deep and moat mats at the A) rRNA and B) rDNA level. The vertical axis is on a log scale. Asterisks indicate signicant differences in abundance of taxa between the deep and moat mat communities (t-test, $p<0.05$).

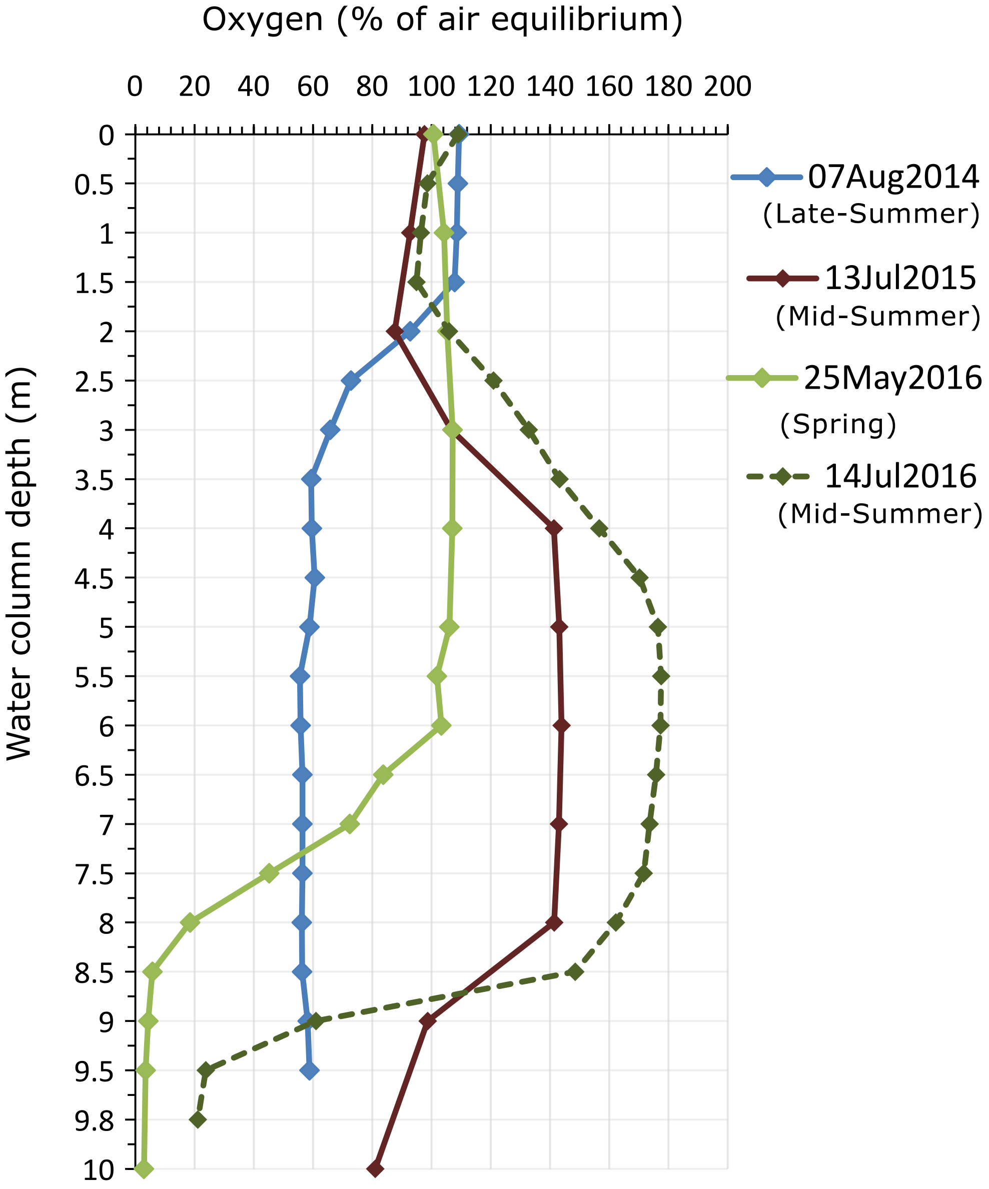

Figure S6: Depth profiles of oxygen saturation (% of air equilibrium) in the deep zone (10 m depth) of Ward Hunt Lake.
